# Supplementary material for: Structural basis for perception of diverse chemical substances by T1r taste receptors
Source: Nat Commun. 2017 May 23;8:15530. doi: 10.1038/ncomms15530 (PMC5457512; doi:10.1038/ncomms15530)
Supplement: Supplementary Information — Supplementary Figures, Supplementary Tables. [file ncomms15530-s1.pdf]

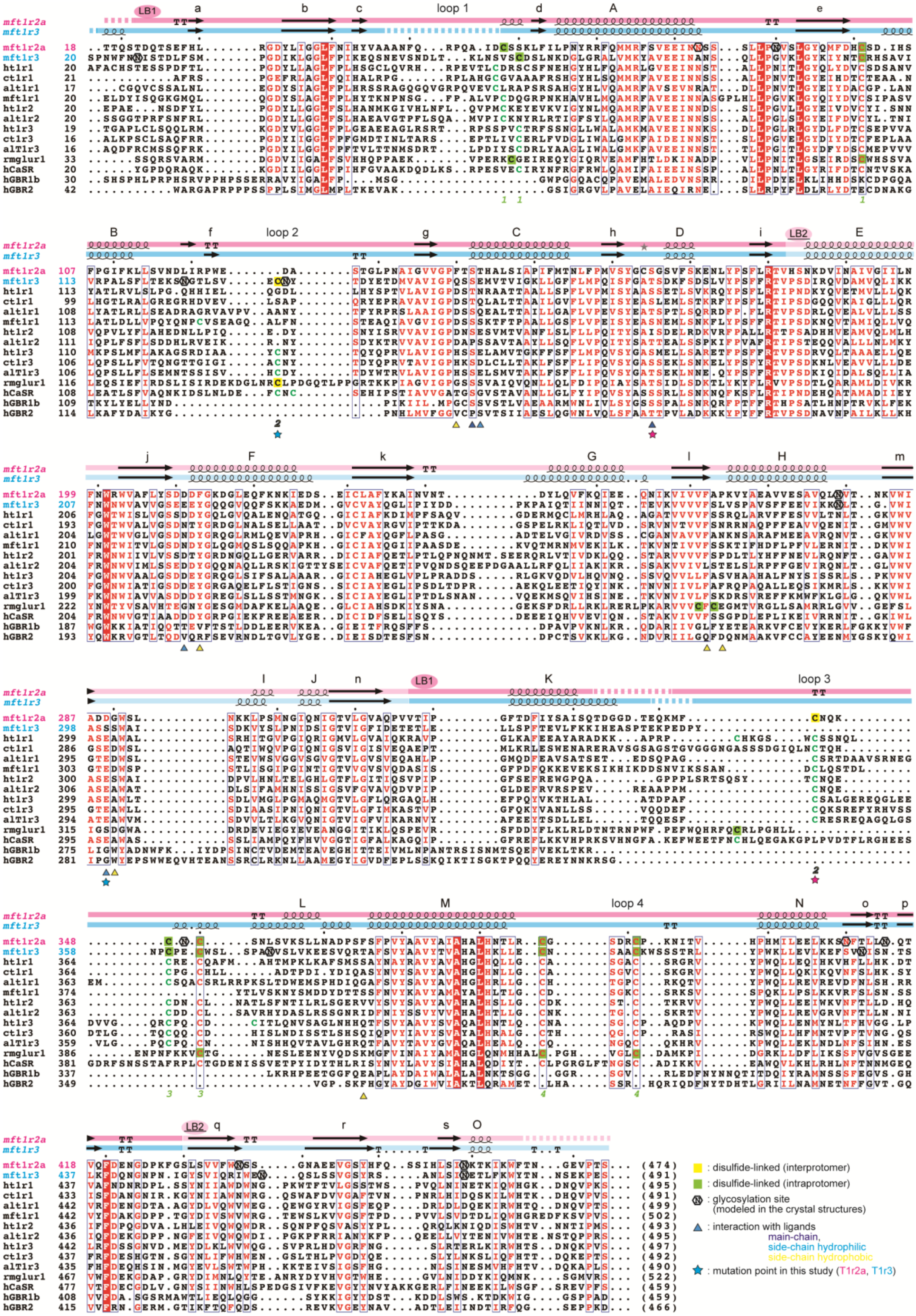

Supplementary Figure 1. Sequence alignment of T1r LBDs. The amino acid sequences of the T1rLBDs from medaka fish (mfT1r1, LC209788; mfT1r2a, BAV00629; mfT1r3,

BAV00630), human (hT1r1, AAI36517; hT1r2, AAM12239; hT1r3, AAI52913), chicken (cT1r1, cT1r3) [7], anole lizard (alT1r1, alT1r2, alT1r3) [7], rat mGluR1 (P23385), human CaSR (AAA86503), human GBR1b (Q9UBS5) and GBR2 (O75899) were aligned. The structure-based amino acid sequence alignment was constructed by PROMALS3D [63], adjusted manually, and drawn by ESPript (http://espript.ibcp.fr, Gouet *et al. Nucleic Acids Res.* **338**, 3320-3323, 2003). The numbers below the sequences pinpoint the sites for interprotomer (yellow) and intraprotomer (green) disulfide bridges. The amino acid residues for glycosylation with obvious electron densities for sugars in the crystal structures, those for ligand interaction, and those subjected to mutation experiments in this study are highlighted.

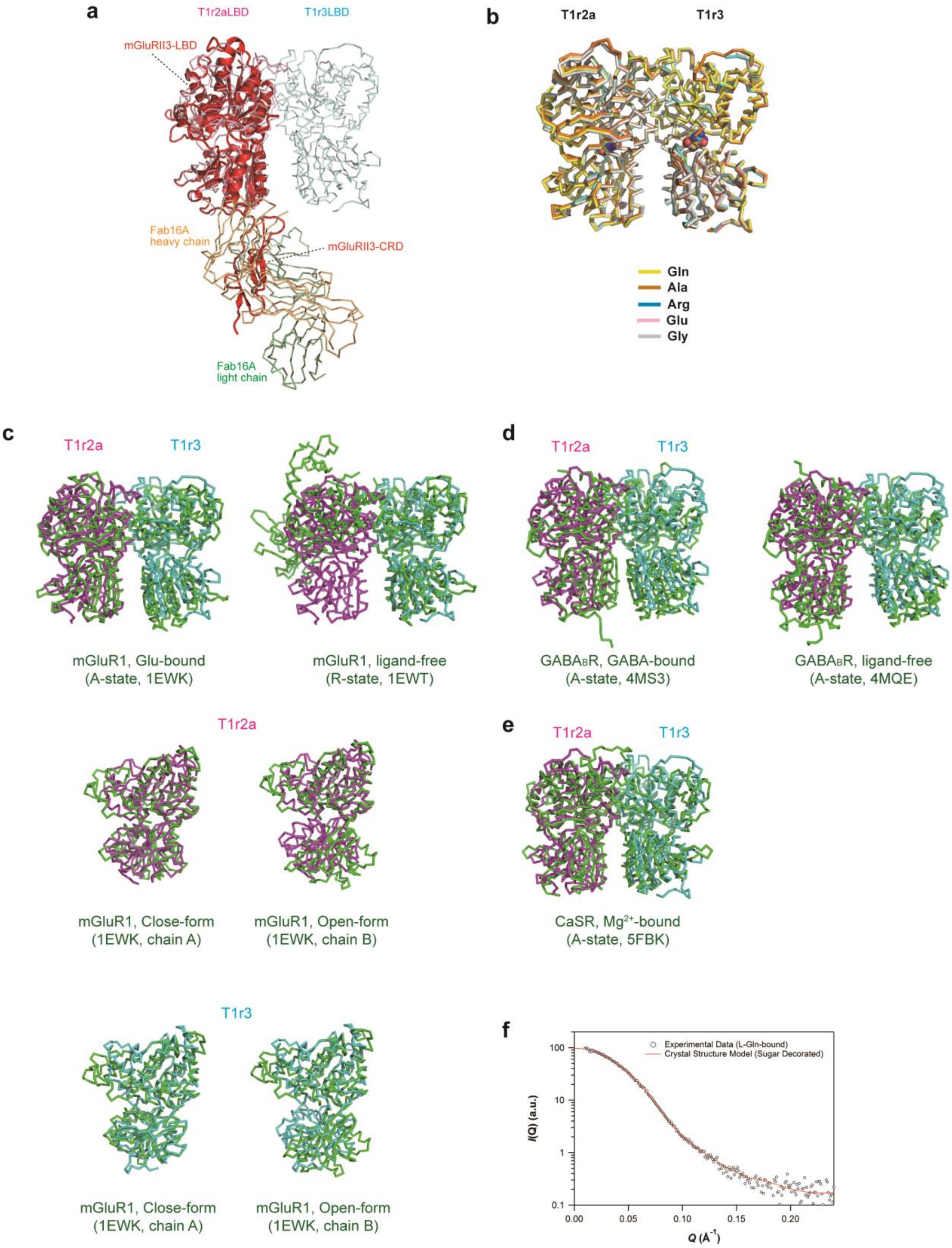

Supplementary Figure 2. The crystal structure of the T1r2a-3LBD heterodimer. (**a**) The structure of the T1r2a-3LBD heterodimer complex with Fab, with superposition of the mGluRII$_3$ extracellular domain, consisting of both the LBD and CRD [46] (as the red ribbon model). The LB2 regions of both structures were used for the secondary-structure matching. The Fab fragment used for crystallization bound to LB2 of T1r2aLBD with the

immunoglobulin fold supplanting the cysteine-rich domain, located downstream of the LBD and upstream of the transmembrane region in the full-length receptor. The bound Fab molecule has no interactions with any regions in the LB1 of T1r2aLBD or the entire T1r3LBD, and thus is unlikely to cause any conformational constraints. (**b**) Comparison of the T1r2a-3LBD structure in complexes with different tastants. The L-glutamine- (yellow), L-alanine- (orange), L-arginine- (blue), L-glutamate- (pink), and glycine-bound (white) T1r2a-3LBD structures are superimposed. (**c-e**) Comparison of the T1r2a-3LBD structure with the extracellular domains of other class C GPCRs. (c) Comparison with mGluR1LBD (PDBID: 1EWK and 1EWT). The comparison with the two different dimerization rearrangements (A- and R-state) and the two different protomer conformation (open- and closed-form) are shown. (d) Comparison with $GABA_B$RLBD (PDBID: 4MSQ and 4MQE). (e) Comparison with CaSRLBD (PDBID: 5FBK). (**f**) Experimental SAXS curve of the L-glutamine-bound T1r2a-3LBD (open circles) compared to theoretical SAXS curve of the crystal structure model with the addition of missing residues and sugar chains (red line) using CRYSOL [60] (Supplementary Table 1). The T1r2a-3LBD crystal structure agreed fairly well with the X-ray solution scattering curve of the glutamine-bound sample (χ = 1.913; Supplementary Table 1), but much more poorly with that of the ligand-free sample (χ = 2.780), analyzed previously [22].

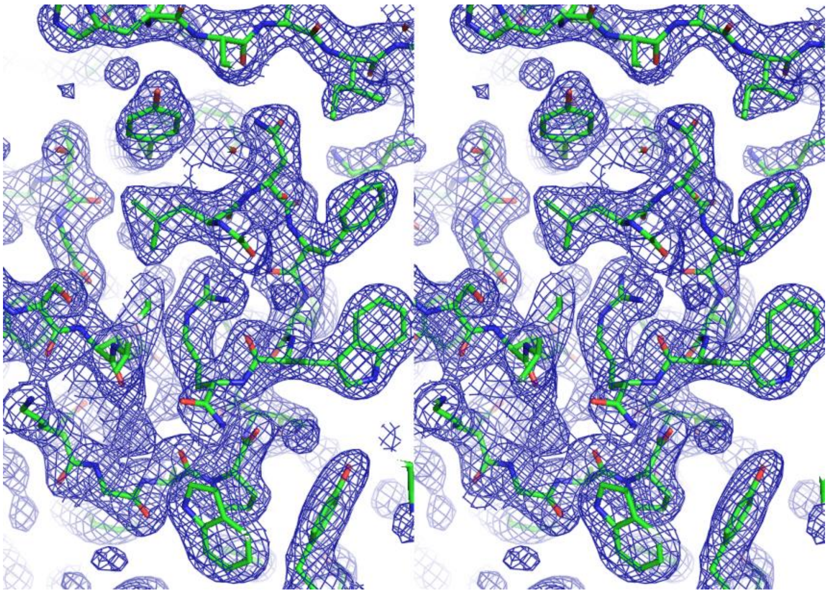

Supplementary Figure 3. Stereo image of the $2F_o - F_c$ electron density map of the glutamine-bound T1r2a-3LBD heterodimer at 1.0 σ.

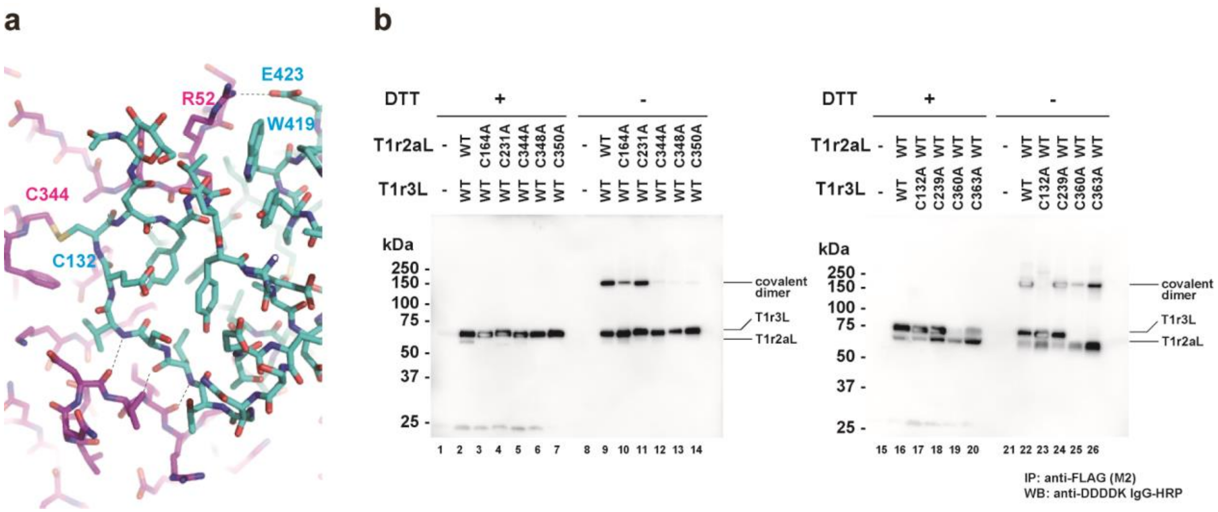

Supplementary Figure 4. Intermolecular disulfide-bridge between T1r2aLBD and T1r3LBD. (**a**) A close-up view of the intermolecular interactions mediated by the loop regions. (**b**) Effects of Ala substitutions of Cys residues on intermolecular disulfide bond formation in T1r2a-3LBD, analyzed by Western blotting. See also Supplementary Fig. 9.

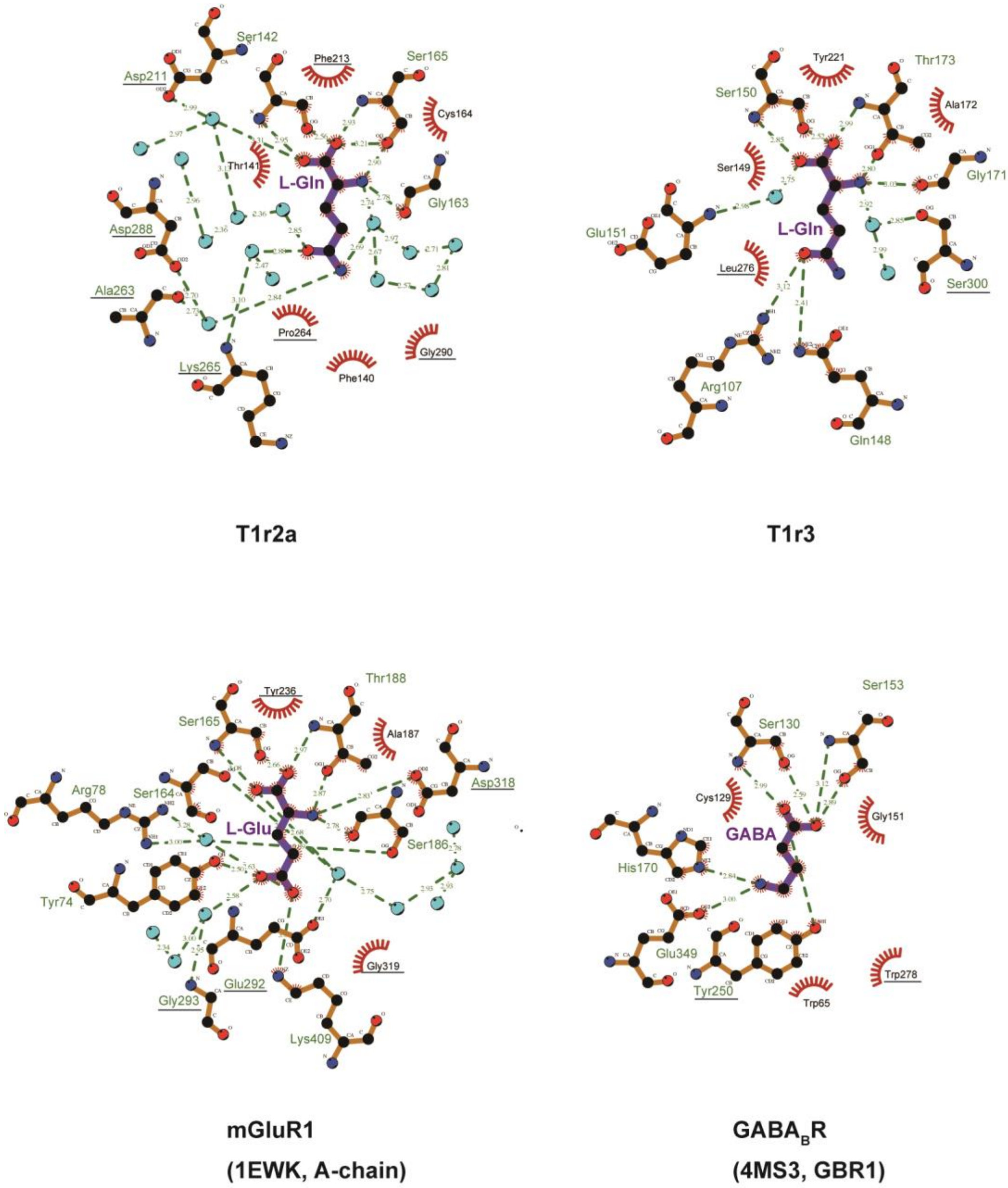

Supplementary Figure 5. Schematic drawings of the receptor-ligand interaction observed in the crystal structures of T1r2aLBD, T1r3LBD, mGluR1LBD (PDB ID: 1EWK), and GABA$_B$RLBD (PDB ID: 4MS3). In all panels, the residues at LB2 are underlined. The figures were generated with LigPlot$^+$ [68].

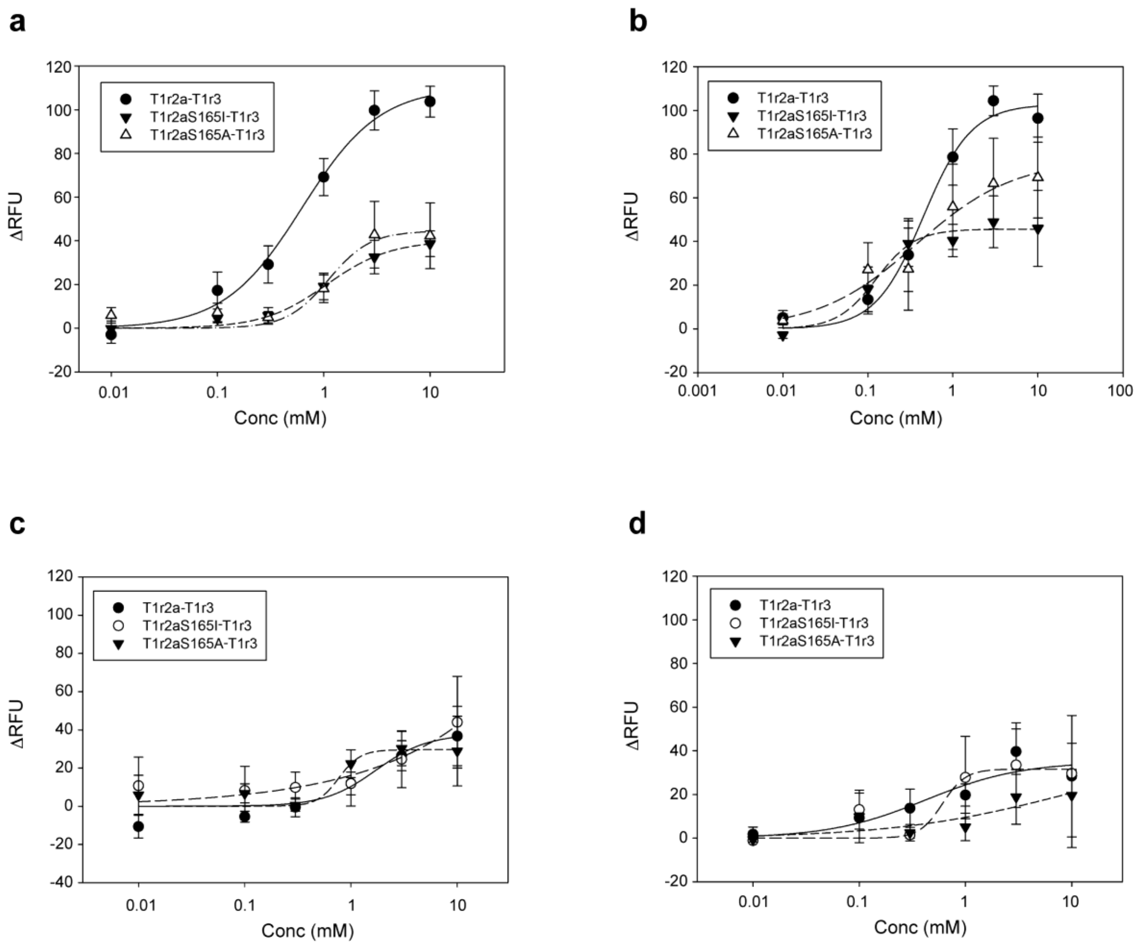

Supplementary Figure 6. Dose-response curves for amino-acids by full-length wild-type T1r2a-T1r3, and T1r2a:S165I-T1r3 and T1r2a:S165A-T1r3 mutant receptors in HEK293 cells, monitored as an elevation of intracellular Ca^{2+} concentration. (**a**) L-alanine responses, (**b**) L-arginine responses, (**c**) D-alanine responses, (**d**) D-arginine responses. Data points represent mean and s.e.m. of 4 technical replicates.

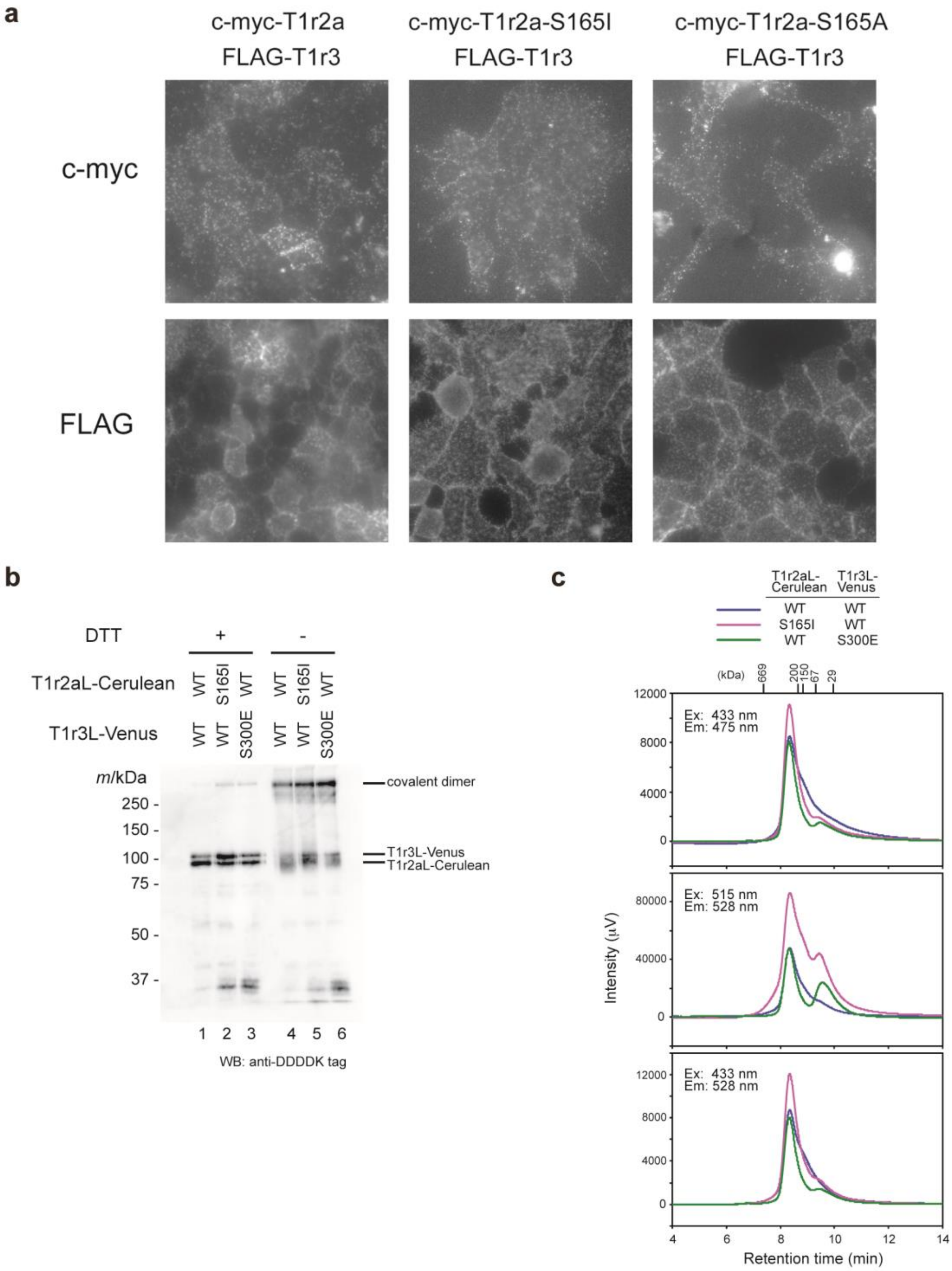

Supplementary Figure 7. Expression properties of the mutant T1r2a-T1r3. (**a**) Cell surface expression of c-Myc-tagged-T1r2a-FLAG-tagged T1r3 in HEK293 cells, labeled with anti-c-Myc (1:200) (polyclonal, Cat No. C3956, Sigma-Aldrich) or anti-FLAG (1:200) (polyclonal, Cat No. F7425, Sigma-Aldrich) antibodies, followed by Alexa Fluor 488-labeled anti-rabbit IgG (1:250) (Cat. No. A-11070, Life Technologies) staining, under non-permeabilized conditions [40]. (**b**) Secreted expression and intermolecular disulfide bond formation of the wild-type and mutant T1r2aLBD-Cerulean and T1r3LBD-Venus heterodimers, analyzed by Western blotting. See also Supplementary Fig. 9. (**c**) Multicolor

fluorescence-detection size-exclusion chromatography (FSEC) [69] profiles of the wild-type and mutant T1r2aLBD-Cerulean and T1r3LBD-Venus heterodimers. The elution profiles analyzed at a specific wavelength pair for the Cerulean detection (top, mainly contributed by T1r2a), Venus detection (middle, mainly by T1r3), or FRET detection (bottom, mainly by heterodimer) are shown. Estimated molecular weight values for the top of the peak of all samples were ~ 240 kDa and roughly agreed with that for T1r2a-Celurean-T1r3-Venus heterodimer (~ 180 kDa, including ~18 kDa glycosylation estimated by previous study [22]).

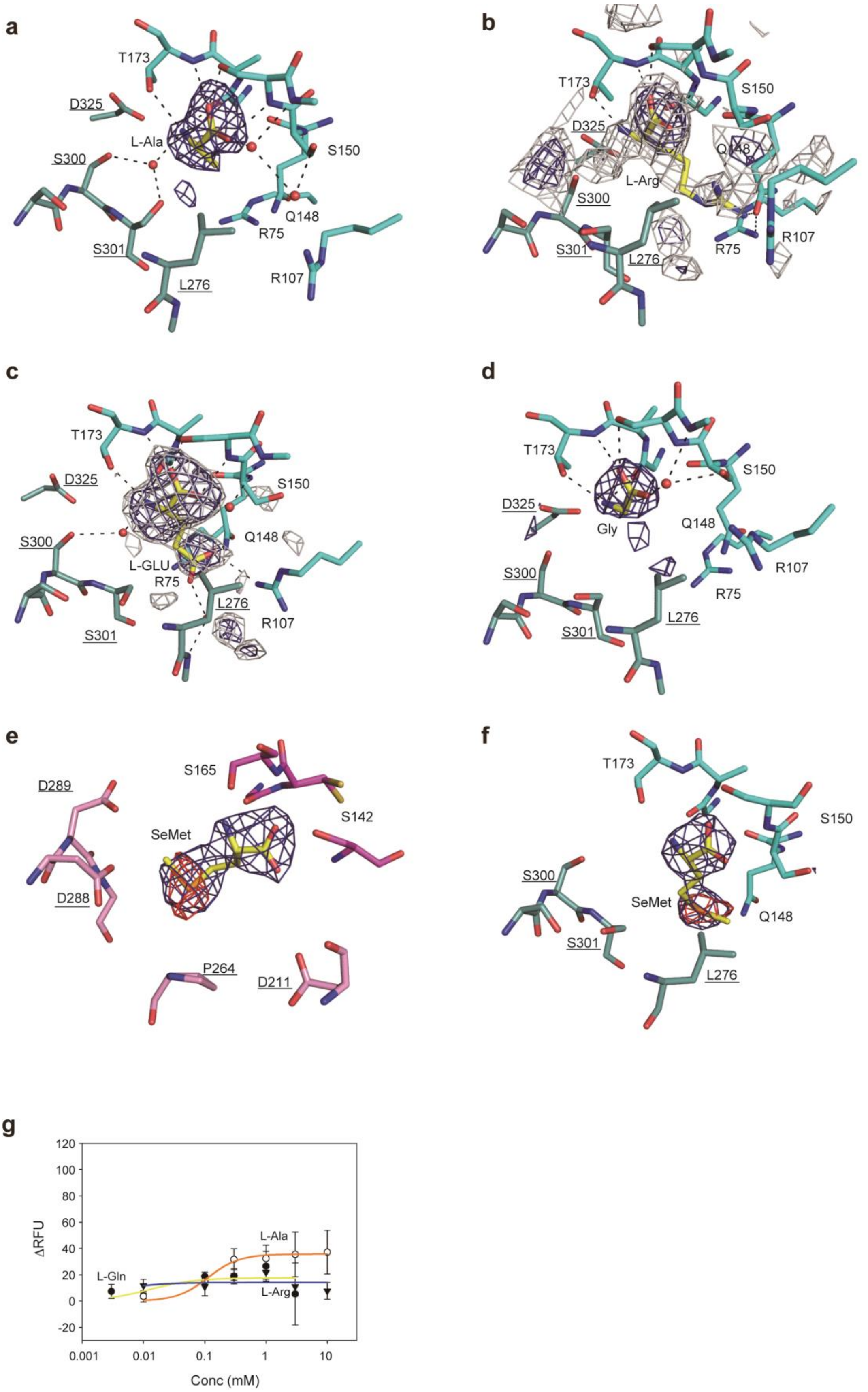

Supplementary Figure 8. The T1r3 ligand-binding site. (**a-d**) L-Alanine (a), L-arginine- (b), L-glutamate- (c), and glycine- (d) bound structures with simulated annealing-omit electron density map at 3.0 σ (blue). In panels b and c, electron density map at 2.2 σ (c) or 1.8 σ (b)

are also shown in gray. (**e**, **f**) Se-anomalous peaks observed at the SeMet-bound T1r2a-3 ligand binding sites. Anomalous difference Fourier map calculated from the data set collected at the wavelength of selenium absorption edge (4.0 σ) is shown in red, and omit map (3.0 σ) is shown in blue. (e) T1r2a site, (f) T1r3 site. In all panels, the residues at LB2 are underlined. (**g**) Dose-response curves for amino-acids by full-length T1r3 alone in HEK293 cells, monitored as an elevation of intracellular Ca^{2+} concentration. Data points represent mean and s.e.m. of 4 technical replicates.

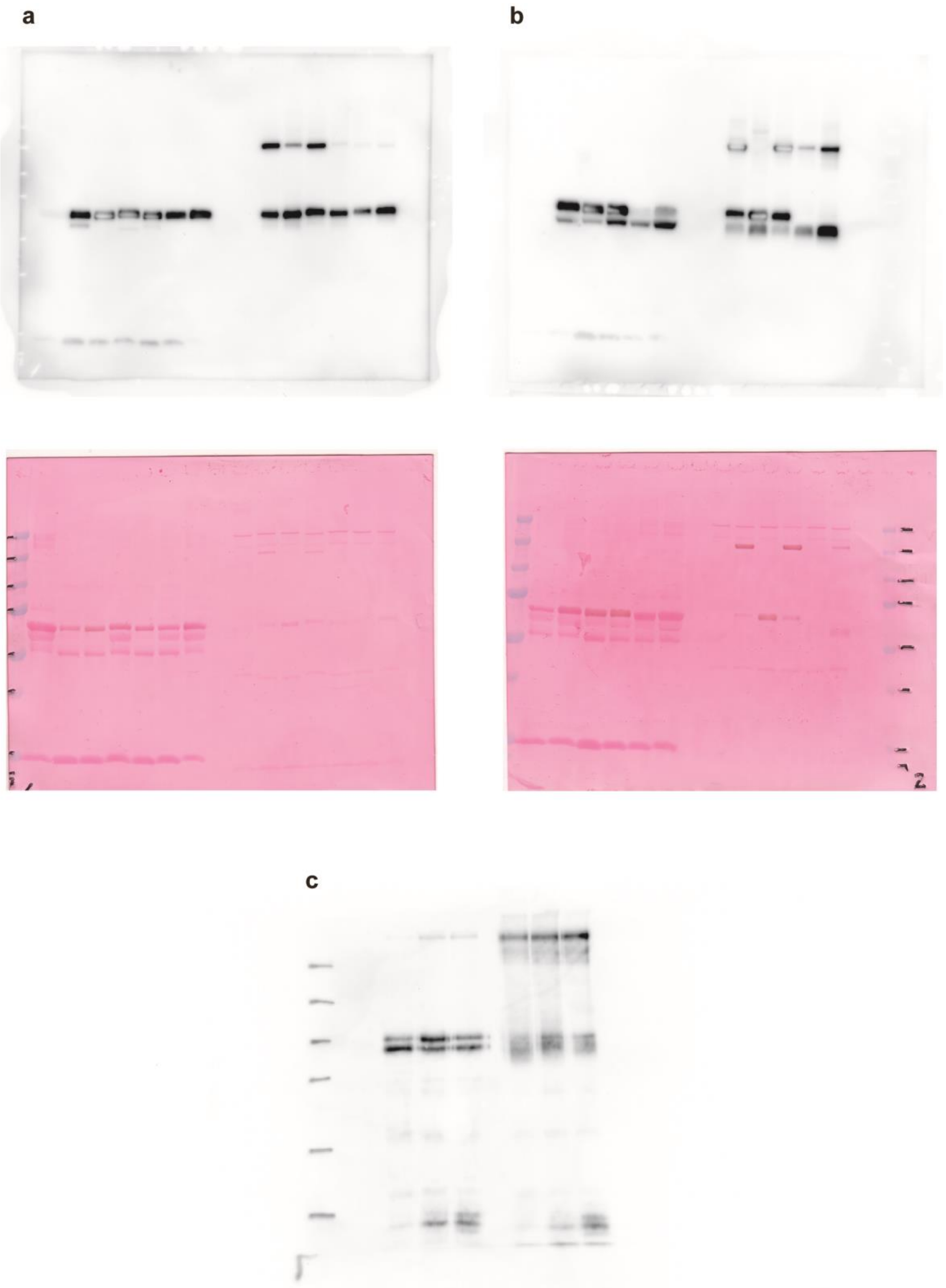

Supplementary Figure 9. Uncropped original images of the blots reported in this study. (**a**, **b**) Images of the blotted membranes shown in Supplementary Fig. 4b. Images detected by chemiluminescence (upper) and Ponceau-S staining (lower) are shown. Panels a and b correspond to the left and right panels in Supplementary Fig. 4b. (b) Image of the blotted membrane detected by chemiluminescence shown in Supplementary Fig. 7b.

Supplementary Table 1. Statistics of the model fitting to the SAXS Data.

	L-Gln-bound SAXS data
Measured	
Q range (Å^{-1})	0.01003 – 0.27683
Real Space Range (Å)	22.7 – 626
CRYSOL	
Q range (Å^{-1})	0.01003 – 0.24072
Real Space Range (Å)	26.1 – 626
Model	
Total Number of Residues	972
Chain A	478
Total Number of Known Residues	446
Known Residues	25 – 470
Total Number of Unknown Residues	32
Unknown Residues	19 – 24, 471 – 496
Chain B	494
Total Number of Known Residues	459
Known Residues	21 – 50, 63 – 491
Total Number of Unknown Residues	35
Unknown Residues	20, 51 – 62, 492 – 513
Total Number of Sugar Chains	21
Chain A	10
Glycosylation Sites	N83, N90, N279, N349, N352, N394, N410, N415, N437, N459
Chain B	11
Glycosylation Sites	N26, N125, N133, N291, N370, N431, N434, N458, N471, N475, N484
Others (Ligands etc.)	L-glutamine
Total Molecular Mass (kDa)	126.5
SQRT (χ^2)	1.912

Supplementary Table 2. EC_{50} values for the receptor response and the FRET signal changes of T1r2a and T1r3 heterodimer to various amino acids.

				FRET changes of LBD (μM)*	Receptor responses (μM)*	
Amino acid	Volume ($Å^3$)†	pI‡	Hydro -pathy index§	T1r2aLBD wt T1r3LBD wt	T1r2a wt T1r3 wt	T1r2a wt T1r3 S300E
L-Glutamine	149.4	5.65	-3.5	11.5 ± 3.4	70±8	200±19
L-Alanine	90.0	6.00	1.8	141 ± 37	2460±2670	1800 ±300
L-Arginine	194.0	10.76	-4.5	190 ± 35	470 ±297	1300±100
L-Glutamate	142.2	3.22	-3.5	1070 ± 382	ND#	ND#
Glycine	64.9	5.97	-0.4	6180 ± 3320	ND#	ND#

*The values are fitted parameters ± s.e. to the Hill equation curves shown in Fig. 2a and 2b. †Tsai *et al. J. Mol. Biol.*, **290**, 253-266,1999. ‡Kyte & Doolittle, *J. Mol. Biol.*, **157**, 105-132, 1982. §Handbook of Chemistry and Physics, CRC Press. #Not determined.

Supplementary Table 3. Sequences of the primers used in this study.

Primers for mutagenesis		
T1r2a C164A	5'-CCTATGGTCAGTTATGGGGCCTCTGGCTCAGTGTTTTCC-3'<br>5'-GGAAAACACTGAGCCAGAGGCCCCATAACTGACCATAGG-3'	
T1r2a C231A	5'-GATTGAAGACAGTGAAATCGCCCTGGCATTCTACAAAGC-3<br>5'-GCTTTGTAGAATGCCAGGGCGATTTCACTGTCTTCAATC-3'	
T1r2a C344A	5'-CACAGAACAGAAAATGTTTGCTAATCAGAAGTGTAACTGC-3'<br>5'-GCAGTTACACTTCTGATTAGCAAACATTTTCTGTTCTGTG-3'	
T1r2a C348A	5'-ATGTTTTGTAATCAGAAGGCTAACTGCAGTAACCTGAGT-3'<br>5'-ACTCAGGTTACTGCAGTTAGCCTTCTGATTACAAAACAT-3'	
T1r2a C350A	5'-TGTAATCAGAAGTGTAACGCCAGTAACCTGAGTGTAAAA-3'<br>5'-TTTTACACTCAGGTTACTGGCGTTACACTTCTGATTACA-3'	
T1r3 C132A	5'-GGGACTCTTTCTGTAGAGGCCAATTACACTGACTATGAG-3'<br>5'-CTCATAGTCAGTGTAATTGGCCTCTACAGAAAGAGTCCC-3'	
T1r3 C239A	5'-GCAGAGGACATGGGTGTTGCTGTGGCCTACCAGGGCTTG-3'<br>5'-CAAGCCCTGGTAGGCCACAGCAACACCCATGTCCTCTGC-3'	
T1r3 C360A	5'-GAGGACCCCTACAACCCCGCTCCAGAGTGCTGGAGCCTG-3'<br>5'-CAGGCTCCAGCACTCTGGAGCGGGGTTGTAGGGGTCCTC-3'	
T1r3 C363A	5'-TACAACCCCTGTCCAGAGGCCTGGAGCCTGTCGCCCGCA-3'<br>5'-TGCGGGCGACAGGCTCCAGGCCTCTGGACAGGGGTTGTA-3'	
T1r2a S165I	5'-GGGTGCATCGGCTCAGTGTTTTCCAAAG-3'<br>5'-TGAGCCGATGCACCCATAACTGACCATAG-3'	
T1r3 S300E	5'-AGAATTTGACCGGTGTTTGGATTGCCAGTGAGAGCTGGGCCATAAGC-3'<br>5'-GCTTATGGCCCAGCTCTCACTGGCAATCCAAACACCGGTCAAATTCT-3'	
**Primers for cloning of the anti- T1r2a-3LBD monoclonal antibody (clone 16A)**		
Heavy chain	5'-GArGTnCAryTrCArCArTCnGGnCCnGAr-3'	degenerate primer of the N-terminal amino acid sequence
	5'-TCATTTACCAGGAGAGTGGGA-3'	constant region primer
Light chain	5'-GAyAThGTnYTnACnCArTCn CCn-3'	degenerate primer of the N-terminal amino acid sequence
	5'-CTAACACTCATTCCTGTTGAA-3	constant region primer
